# Bounds for the Diameters of Orbital Graphs of Affine Groups

**DOI:** 10.1007/s10013-023-00607-5

**Published:** 2023-02-10

**Authors:** Attila Maróti, Saveliy V. Skresanov

**Affiliations:** 1 Alfréd Rényi Institute of Mathematics, Reáltanoda utca 13-15, 1053 Budapest, Hungary; 2 Sobolev Institute of Mathematics, 4 Acad. Koptyug avenue, Novosibirsk, Russia

**Keywords:** Orbital graph, Diameter, Affine primitive permutation group, 20B15, 20F69

## Abstract

General bounds are presented for the diameters of orbital graphs of finite affine primitive permutation groups. For example, it is proved that the orbital diameter of a finite affine primitive permutation group with a nontrivial point stabilizer *H* ≤GL(*V* ), where the vector space *V* has dimension *d* over the prime field, can be bounded in terms of *d* and $\log |V|/\log |H|$ only. Several infinite families of affine primitive permutation groups with large orbital diameter are constructed. The results are independent from the classification of finite simple groups.

## Introduction

Connections between finite permutation groups and graphs quite often allow one to express some abstract group-theoretic properties in combinatorial terms. One of the classical approaches is the study of orbital graphs of permutation groups. Recall that if *G* is a permutation group acting on a finite set *X*, then an *orbital graph* of *G* is a graph with vertex set *X* whose arc set is an orbit of *G* on *X* × *X*. An orbital graph whose arcs are a subset of the diagonal {(*x*,*x*) | *x* ∈ *X*} is called a *diagonal* orbital graph.

A transitive permutation group is called *primitive* if it has no nontrivial proper block of imprimitivity or, equivalently, if a point stabilizer is a maximal subgroup in the group. Primitive permutation groups can also be characterized in terms of their orbital graphs. By a criterion of Higman [11], a finite permutation group is primitive if and only if all its non-diagonal orbital graphs are connected. The degree of an orbital graph of a finite primitive permutation group is also of fundamental importance. By the Sims conjecture, resolved by Cameron, Praeger, Saxl and Seitz [7] (see also a simplified proof by Pyber and Tracey [18]) if some non-diagonal orbital graph of a finite primitive permutation group has degree bounded by *d*, then a point stabilizer of the group has size bounded in terms of *d*.

The subject of this paper is the diameter of an orbital graph of a finite primitive permutation group, or *orbital diameter*. Using model theory, Liebeck, Macpherson and Tent [15] described finite primitive permutation groups whose non-diagonal orbital graphs have bounded diameter (we note that in [15] orbital graphs are considered to be undirected), see also the papers of Sheikh [20] and Rekvényi [19]. The converse problem of finding upper bounds on the diameters of orbital graphs of primitive groups was also considered in [15], but in the case of affine groups only a partial result was obtained, see [15, Lemma 3.1].

Recall that a finite primitive permutation group *G* acting on a set *X* is called *affine* if it contains a nontrivial normal subgroup *V* which is elementary abelian (and regular as a permutation group). In this case a stabilizer *H* in *G* of a point in *X* acts linearly on *V*, viewed as a vector space over the prime field $\mathbb {F}_{p}$, where *p* is prime. Moreover *V* is a faithful and irreducible $\mathbb {F}_{p}H$-module and conversely, if *V* is a faithful and irreducible $\mathbb {F}_{p}H$-module for a finite group *H*, then the semidirect product *HV* may be viewed as an affine primitive permutation group with socle *V*. The main goal of this paper is to provide detailed upper bounds on the diameters of orbital graphs of affine primitive permutation groups, and complement the results of [15].

Another motivation is a problem of finding a *n*^*o*(1)^ upper bound on diameters of orbital graphs of some classes of primitive permutation groups of degree *n* and, more generally, a bound on diameters of constituent graphs of primitive coherent configurations. See Pyber’s paper [17] proving a logarithmic bound for distance-regular graphs and his conjecture in [14, p. 119]. We show that there exist several infinite families of affine primitive permutation groups of degree *n* = *p*^*d*^, where *p* is a prime, with orbital diameter at least (*p* − 1)*d*/4 (see Section 7). In particular, the *n*^*o*(1)^ bound is not achievable for affine groups. We note that a weaker version of this was found earlier by Pyber (personal communication).

Our third motivation comes from additive combinatorics. Cochrane and Cipra [8, Theorem 1.2] proved the following result on the Waring problem in finite fields. Let *A* be a nontrivial multiplicative subgroup of *F*^×^, where *F* is a finite field, and assume that *A* generates *F* additively. Then *n* ⋅ *A* = *F* for every $ n \geq 633 \cdot |F|^{\frac {\log 4}{\log |A|}} $, where *n* ⋅ *A* denotes the sum of *n* copies of the set *A* (see Proposition 4.2 for a more precise statement), in particular, the diameter of a non-diagonal orbital graph of the affine primitive permutation group *AF* is bounded by *n*. Another goal of this paper is to present a generalization of this theorem (see Theorem 1.1).

The theorem of Cochrane and Cipra should be compared to the following results of Babai. Let *r* and *p* be primes such that *r* divides *p* − 1 and let *H*(*p*,*r*) be the unique nonabelian group of order *pr*. This group can be generated by two elements. Let $\text {diam}_{\min \limits }(H(p,r))$ and $\text {diam}_{\max \limits }(H(p,r))$ denote the minimum and, respectively, maximum diameter of a Cayley graph of *H*(*p*,*r*) over all possible pairs of generators. It was shown by Babai in an unpublished work that if *r* is bounded and $p \to \infty $ then both $\text {diam}_{\min \limits }(H(p,r))$ and $\text {diam}_{\max \limits }(H(p,r))$ have the order of *Θ*(*p*^1/(*r*− 1)^) (see [3, Theorem 6.1]). If *r* > *p*^1/2+*c*^ for some constant *c* > 0 then $\text {diam}_{\min \limits }(H(p,r)) = {\varTheta }(r^{1/2})$ and $\text {diam}_{\max \limits }(H(p,r)) = {\varTheta }(r)$ (see [3, Theorem 6.2]). The second result is connected with the Waring problem in $\mathbb {F}_{p} $, see [3, Section 6] for details.

Another related result is a theorem of Peluse [16] on exponential sums over orbits of a linear group. It was shown that for every positive integer *d* and positive numbers *δ*, *β* there is a positive *𝜖* such that whenever *HV* is an affine primitive permutation group where *H* ≤GL(*V* ) and *V* is a vector space of dimension *d* over $\mathbb {F}_{p}$ and *Δ* is a nonzero orbit of *H* on *V* with (1) |*Δ*|≥ *p*^*δ*^ and (2) |*Δ*∩ *P*|≤|*Δ*|^1−*β*^ for every hyperplane *P* in *V*, then the absolute value of an exponential sum over *Δ* is bounded by *p*^−*𝜖*^|*Δ*|. By Fourier analysis, this bound implies that the maximum diameter of a non-diagonal orbital graph of the affine primitive permutation group *HV* is bounded in terms of *d*, *δ* and *β* only. The third goal of the paper is to show that the maximum diameter of a non-diagonal orbital graph of an affine primitive permutation group is bounded in terms of *d* and *δ* provided that condition (1) holds (see Corollary 1.2).

To state our main result, let *V* be a faithful irreducible $\mathbb {F}_{p}H$-module for a finite group *H*, and let *d* be the dimension of *V* over $ \mathbb {F}_{p} $. Denote by $ \overrightarrow {\text {diam}}(V, H) $ the supremum of the diameters of non-diagonal orbital graphs of *HV* where graphs are considered to be oriented, and let diam(*V*,*H*) be the supremum of diameters of non-diagonal orbital graphs where we forget about arc directions and consider graphs to be undirected. It follows from elementary diameter estimates (see Propositions 3.1 and 3.2) that

$$ \frac{1}{2}(|V|^{1/|H|}-1) \leq \text{diam}(V, H) \leq \overrightarrow{\text{diam}}(V, H) \leq (p-1)d. $$

Our main result may be considered an improvement of the previous upper bound on the diameter in the case when *p* is large.

### **Theorem 1.1**

Let *HV* ≤AGL(*V* ) be an affine primitive permutation group with *V* a vector space of dimension *d* over $\mathbb {F}_{p}$, *p* prime, and *H* a point stabilizer.

1. If *H* has a composition factor isomorphic to a finite simple group of Lie type in characteristic *p*, then
  $$ \text{diam}(V,H) < 2^{22d^{3}}, $$
  in particular, the diameter is bounded in terms of $\dim \text {V}$ only.
2. There exists a function *J*(*d*) depending only on *d* such that whenever *H* does not have a composition factor isomorphic to a finite simple group of Lie type in characteristic *p*, and |*H*|≥ *J*(*d*)^2^, then
  $$ \overrightarrow{\text{diam}}(V,H) < 2^{18d^{2}} \cdot {|V|}^{\frac{d\log 64}{\log |H|}}. $$

Part (2) of Theorem 1.1 may be viewed as a generalization of the aforementioned theorem of Cochrane and Cipra [8] for arbitrary affine primitive groups in the case when *d* is bounded. Notice that in part (1) we are able to obtain an upper bound on undirected diameters only. In general, $\overrightarrow {\text {diam}}(V,H) < 18d \cdot \log p \cdot \text {diam}(V,H)$ by [2, Theorem 1.5] since orbital graphs are edge-transitive. It might be possible to obtain an upper bound on $\overrightarrow {\text {diam}}(V, H)$ in terms of *d* only in our case, but the technique employed by the authors does not allow that.

The function *J*(*d*) from the statement of (2) of Theorem 1.1 is precisely the function from the Larsen and Pink theorem, see Proposition 5.2, which is a classification-free version of Weisfeiler’s theorem on the structure of linear groups [22, 23]. We will show in Section 7 that there exist groups *H* of size bounded in terms of *d* with orbital graphs of large diameter, so the condition that |*H*| is larger than some constant depending on *d* is essential in (2) of Theorem 1.1.

In [15, Part (1) of Theorem 1.1] it was shown that if a class of affine primitive permutation groups has bounded orbital diameter, then this class consists of the so-called groups of *t*-bounded classical type, for some bounded *t*. It follows from the definition of groups of *t*-bounded classical type that an affine group *HV* with $ d = \dim V $ is of *t*-bounded classical type for all *t* ≥ *d*, and therefore the conclusion of [15, Theorem 1.1] is nontrivial only for classes of groups with unbounded *d* (see the remark before [15, Lemma 3.2]).

As a corollary to Theorem 1.1, one can show that diameters are controlled by the ratio $ \log |V| / \log |H| $, when *d* is bounded.

### **Corollary 1.2**

For any *d* there exists a constant *f*(*d*) depending on *d* only such that for any affine primitive permutation group *HV* ≤AGL(*V* ) with *V* a vector space of dimension *d* over $\mathbb {F}_{p}$ and 1 < *H* ≤GL(*V* ), we have

$$ \frac{\log |V|}{3\log |H|} \leq \text{diam}(V,H) \leq f(d)^{\frac{\log |V|}{\log |H|}}. $$

In particular, when *d* is bounded, diam(*V*,*H*) is bounded if and only if $\frac {\log |V|}{\log |H|} $ is bounded.

In Section 7 we will show that *f*(*d*) grows at least quasipolynomially.

In order to prove Theorem 1.1 we establish the following technical result providing a more precise upper bound on the diameters of orbital graphs. Recall that a finite group is called a $ p^{\prime } $-group if its order is not divisible by a prime *p*.

### **Theorem 1.3**

Let *HV* ≤AGL(*V* ) be an affine primitive permutation group where *V* is a vector space of dimension *d* over $\mathbb {F}_{p}$ and *H* ≤GL(*V* ) acts irreducibly on *V*. Let *A* be a nontrivial abelian $p^{\prime }$-subgroup of *H* and let *k* be the number of irreducible summands of the completely reducible $\mathbb {F}_{p}A$-module *V*. Then

$$ \text{diam}(V,H) < 322 d \cdot 144^{k(k+1)} \cdot {|V|}^{\frac{k (k+1)\log 4}{\log |A|}}. $$

Moreover, if *A* is normal in *H*, then

$$ \overrightarrow{\text{diam}}(V,H) < d \cdot 2576^{k(k+1)} \cdot {|V|}^{\frac{(k+1)\log 4}{\log |A|}}. $$

The proof of Theorem 1.3 relies on the result of Cochrane and Cipra [8, Theorem 1.2].

It should be mentioned that our results are free from the classification of finite simple groups. By utilizing the classification it is possible to give a good bound on the function *J*(*d*) (see, for example, the bounds in [23] or [9]) and improve the upper bounds in the case when the group contains a composition factor isomorphic to a finite simple group of Lie type in characteristic *p*, but these questions are out of scope of this paper.

The structure of the paper is as follows. In Section 2 we provide necessary preliminaries on additive properties of subsets and reformulate the problem of bounding diameters in those terms. In Section 3 we give elementary upper and lower bounds on the diameters, in particular, we provide an upper bound in terms of the intersection of the point stabilizer with the group of scalar matrices. Section 4 contains the proof of Theorem 1.3. In Section 5 we derive Theorem 1.1 from Theorem 1.3, and in Section 6 we derive Corollary 1.2. In Section 7 we provide examples of affine primitive permutation groups with large diameter, proving that some of our estimates are tight.

## Preliminaries

Let *V* be a finite abelian group, and let *Δ*, *Γ* be subsets of *V*. We define the sum, difference and negation of subsets as usual:

$$ \begin{array}{@{}rcl@{}} {\varDelta} + {\varGamma} &=& \{x + y~|~x \in {\varDelta}, y \in {\varGamma} \},\\ {\varDelta} - {\varGamma} &=& \{ x - y ~|~ x \in {\varDelta}, y \in {\varGamma} \},\\ - {\varDelta} &=& \{ -x ~|~ x \in {\varDelta} \}. \end{array} $$

For an integer *n* ≥ 1 let *n* ⋅*Δ* denote the sum of *n* copies of *Δ*. The following proposition records several properties of subset sums, which will be used without further notice.

### **Proposition 2.1**

Let *V* be a finite abelian group, and let ${\varDelta } \subseteq V$.

1. If *m* ≤ *n*, then |*m* ⋅*Δ*|≤|*n* ⋅*Δ*|. Furthermore, if 0 ∈*Δ*, then $ m \cdot {\varDelta } \subseteq n \cdot {\varDelta } $.
2. If *m* ⋅*Δ* = *V*, then for any *n* ≥ *m* we have *n* ⋅*Δ* = *V*.
3. If $ {\varDelta } + {\varDelta } \subseteq {\varDelta } $ and *Δ* is nonempty, then *Δ* is a subgroup of *V*.

### *Proof*

If *Δ* is nonempty and *x* ∈*Δ*, then for any $ {\varGamma } \subseteq V$ we have

$$ |{\varGamma}| = |{\varGamma} + x| \leq |{\varGamma} + {\varDelta}|, $$

where the last inequality holds since $ {\varGamma } + x \subseteq {\varGamma } + {\varDelta }$. Furthermore, if 0 ∈*Δ*, then $ {\varGamma } \subseteq {\varGamma } + {\varDelta } $, and (1) follows.

Property (2) is a direct consequence of (1), and (3) is well-known for arbitrary finite groups.□

Let *G* be a permutation group acting on a finite set *X*. An orbital graph for (*X*,*G*) is a graph with vertex set *X* whose arc set is an orbit of *G* on *X* × *X*; in general, this is a directed graph. An orbital graph with edge set contained in {(*x*,*x*) : *x* ∈ *X*} is called a diagonal orbital graph. The criterion of Higman [11] states that a permutation group *G* acting on *X* is primitive if and only if all non-diagonal orbital graphs are (strongly) connected, see [6, Theorems 1.9 and 1.10] for the proof.

Assume that *G* is an affine primitive permutation group with socle *V*. The group *V* is elementary abelian and acts regularly on *X*, so it can be identified with *X* in a natural way. Viewing *V* as a vector space of dimension *d* over the finite field $\mathbb {F}_{p}$ the group *G* can be considered a subgroup of the affine general linear group AGL(*V* ). Therefore *G* decomposes as a semidirect product *HV* where *H* is the stabilizer of 0 ∈ *V*, and *V* is a faithful irreducible $\mathbb {F}_{p}H$-module.

In the case of an affine permutation group one can easily see that its orbital graphs are Cayley graphs of an abelian group *V*. Indeed, if *Δ* is an orbit of *H* on *V*, then the corresponding orbital graph has arc set {(*x*,*y*) ∈ *V* × *V* | *x* − *y* ∈*Δ*}, so *Δ* is the connection set. Notice that we obtain the diagonal orbital graph when *Δ* is the zero orbit.

If $ x_{1}, \dots , x_{k} \in V $ is a directed path in an orbital graph corresponding to the orbit *Δ* of *H* on *V*, then $ x_{1} - x_{2} \in {\varDelta }, x_{2} - x_{3} \in {\varDelta }, \dots , x_{k-1} - x_{k} \in {\varDelta } $, and therefore *x*_1_ − *x*_*k*_ ∈ (*k* − 1) ⋅*Δ*. It follows that the (directed) diameter of the corresponding orbital graph is equal to the minimal number *n* ≥ 1 such that

$$ \{ 0 \} \cup (1 \cdot {\varDelta}) \cup (2 \cdot {\varDelta}) \cup {\dots} \cup (n \cdot {\varDelta}) = V, $$

or, in other words, to the minimal *n* ≥ 1 such that *n* ⋅ (*Δ* ∪{0}) = *V*. Observe that such *n* always exists for a nonzero orbit *Δ*, since *H* acts irreducibly on *V* and therefore *Δ* spans *V* over $ \mathbb {F}_{p} $.

Denote by $\overrightarrow {\text {diam}}(V, H)$ the supremum of the diameters of non-diagonal orbital graphs of (*X*,*G*). Let $ {\varDelta }_{1}, \dots , {\varDelta }_{r} $ be all the nonzero orbits of *H* on *V*. Then

1 $$ \overrightarrow{\text{diam}}(V, H) = \min \{ n \in \mathbb{N} ~|~ n \cdot ({\varDelta}_{i} \cup \{0\}) = V \text{ for all } i = 1, \dots, r \}. $$

If we forget about the arc direction of an orbital graph, we can consider its undirected diameter; let diam(*V*,*H*) denote the supremum of the undirected diameters of non-diagonal orbital graphs of (*X*,*G*). If *Δ* is the connection set of some orbital graph, then the corresponding undirected graph has connection set *Δ* ∪−*Δ*, in particular, we obtain the formula

2 $$ \text{diam}(V, H) = \min \{ n \in \mathbb{N} ~|~ n \cdot ({\varDelta}_{i} \cup -{\varDelta}_{i} \cup \{ 0 \}) = V \text{ for all } i = 1, \dots, r \}. $$

Clearly the orbits of the group *H*〈− 1〉 on *V* are *Δ*_*i*_ ∪−*Δ*_*i*_, $i = 1, \dots , r$, so $\text {diam}(V, H) = \overrightarrow {\text {diam}}(V, H\langle -1 \rangle )$.

The following lemma shows that if all elements of some nontrivial subspace lie at distance at most *m* from 0 in an orbital graph, then the whole orbital graph has diameter at most *dm*, where *d* is the dimension of *V*.

### **Lemma 2.2**

Let *V* be a vector space of dimension *d* over the finite field $\mathbb {F}_{p}$ of order *p*. Let *H* be a finite group acting irreducibly on *V*. For any subgroup *A* of *H*, any nontrivial subspace *W* of *V* and any nonzero vector *v* in *V*, if $W \subseteq m \cdot (v^{A} \cup \{0\})$ for some *m*, then *dm* ⋅ (*v*^*H*^ ∪{0}) = *V*.

### *Proof*

Let *u* ∈ *W* be a nonzero vector. The orbit *u*^*H*^ spans *V* over $\mathbb {F}_{p}$, so there exist elements $h_{1}, \dots , h_{d} \in H$ such that $u^{h_{1}}, \dots , u^{h_{d}}$ is a basis of *V* over $\mathbb {F}_{p}$. Since $\mathbb {F}_{p} u \subseteq W \subseteq m \cdot (v^{A} \cup \{0\})$ and *A* ≤ *H*, we have

$$ \mathbb{F}_{p}u^{h_{1}}, \dots, \mathbb{F}_{p}u^{h_{d}} \subseteq m \cdot (v^{H} \cup \{ 0 \}). $$

Therefore $V = \mathbb {F}_{p}u^{h_{1}} + {\dots } + \mathbb {F}_{p}u^{h_{d}} \subseteq dm \cdot (v^{H} \cup \{0\})$, and the claim is proved.□

By considering groups *H*〈− 1〉 and *A*〈− 1〉 we obtain an undirected version of the above result: if $ W \subseteq m \cdot (v^{A} \cup -v^{A} \cup \{0\})$ for some *m*, then *dm* ⋅ (*v*^*H*^ ∪−*v*^*H*^ ∪{0}) = *V*.

## Elementary Diameter Estimates

Let *HV* ≤AGL(*V* ) be an affine primitive permutation group where *V* is a vector space of dimension *d* over the prime field $\mathbb {F}_{p}$ and *H* ≤GL(*V* ). First we prove two general lower bounds for the diameter. We follow the proof of [4, Proposition 1.1] for our second lower bound, see also [1, Theorem 3].

### **Proposition 3.1**

For nontrivial *H* let *s* be the size of the smallest nonzero orbit of *H* on *V*. Then

$$ \frac{\log |V|}{3\log |H|} \leq \frac{\log |V|}{\log (2s+1)} \leq \text{diam}(V, H) $$

and

$$ \frac{1}{2}(|V|^{1/|H|}-1) \leq \frac{1}{2}(|V|^{1/s}-1) \leq \text{diam}(V, H). $$

### *Proof*

Let *Δ* be a nonzero orbit of *H* on *V* of size *s*. Set *n* = diam(*V*,*H*) and recall that *n* ⋅ (*Δ* ∪−*Δ*∪{0}) = *V*. Therefore

$$ |V| = |n \cdot ({\varDelta} \cup -{\varDelta} \cup \{ 0 \})| \leq |{\varDelta} \cup -{\varDelta} \cup \{ 0 \}|^{n} \leq (2s+1)^{n}, $$

and the first displayed inequalities are proved.

Set ${\varDelta } = \{x_{1}, \dots , x_{s}\}$. Then every vector *x* ∈ *V* can be expressed as the sum *x* = *k*_1_*x*_1_ + ⋯ + *k*_*s*_*x*_*s*_, where |*k*_*i*_|≤ *n*. Therefore |*V* |≤ (2*n* + 1)^*s*^, and the second result follows. □

Since $\text {diam}(V, H) \leq \overrightarrow {\text {diam}}(V, H)$, the provided bounds are also lower bounds for the directed diameter.

Let $\mathbb {F}_{p}^{\times }$ denote the multiplicative group of the finite field $\mathbb {F}_{p}$, and recall that we can identify $\mathbb {F}_{p}^{\times }$ with the center of GL(*V* ) in a natural way. The next result shows that the diameter is controlled by the intersection of the group *H* with $\mathbb {F}_{p}^{\times }$, essentially generalizing [15, Part (i) of Lemma 3.1].

### **Proposition 3.2**

We have

$$ \overrightarrow{\text{diam}}(V,H) \leq \overrightarrow{\text{diam}}(\mathbb{F}_{p},\mathbb{F}_{p}^{\times} \cap H) \cdot d \leq (p-1)d $$

for all *p*, and

$$ \text{diam}(V,H) \leq \text{diam}(\mathbb{F}_{p},\mathbb{F}_{p}^{\times} \cap H\langle -1 \rangle) \cdot d \leq (p-1)d/2, $$

when *p* is odd.

### *Proof*

Set $A = \mathbb {F}_{p}^{\times } \cap H$ and let *v* ∈ *V* be a nonzero vector. Since *A* is a nonzero orbit of *A* acting on $\mathbb {F}_{p}$ by right multiplication, we have $n \cdot (A \cup \{ 0 \}) = \mathbb {F}_{p}$, where $n = \overrightarrow {\text {diam}}(\mathbb {F}_{p}, A)$. Therefore *n* ⋅ (*v*^*A*^ ∪{0}) is a nontrivial subgroup of *V*, and by Lemma 2.2, we have *dn* ⋅ (*v*^*H*^ ∪{0}) = *V*. Thus $\overrightarrow {\text {diam}}(V, H) \leq \overrightarrow {\text {diam}}(\mathbb {F}_{p}, A) \cdot d$. Now, $\overrightarrow {\text {diam}}(\mathbb {F}_{p}, A) \leq \overrightarrow {\text {diam}}(\mathbb {F}_{p}, \langle 1 \rangle )$. Since non-diagonal orbital graphs of the trivial group acting on $\mathbb {F}_{p}$ are directed cycles of length *p*, we obtain $\overrightarrow {\text {diam}}(\mathbb {F}_{p}, \langle 1 \rangle ) = p-1$ and the first displayed inequalities are proved.

To prove the second part, recall that $\text {diam}(V, H) = \overrightarrow {\text {diam}}(V, H\langle -1 \rangle )$, hence $\text {diam}(V, H) \leq \text {diam}(\mathbb {F}_{p}, \mathbb {F}_{p}^{\times } \cap H\langle -1 \rangle ) \cdot d$ by the previous paragraph. Clearly $\text {diam}(\mathbb {F}_{p}, \mathbb {F}_{p}^{\times } \cap H\langle -1 \rangle ) \leq \text {diam}(\mathbb {F}_{p}, \langle -1 \rangle )$. Finally, we have $\text {diam}(\mathbb {F}_{p}, \langle -1 \rangle ) = (p-1)/2$ for odd *p*, since orbital graphs of 〈− 1〉 acting on $\mathbb {F}_{p}$ are undirected cycles of length *p*.□

In Section 7 we will show that for all *d* and all odd *p* the inequalities on undirected diameter presented in Proposition 3.2 are sharp. Notice that the bound on undirected diameter does not apply when *p* = 2, as $\text {diam}(\mathbb {F}_{2}, \langle 1 \rangle ) = 1$.

## Proof of Theorem 1.3

In this section we prove Theorem 1.3 which is the main technical result required for the proof of Theorem 1.1.

Let *H* be an irreducible subgroup of GL(*V* ), where *V* has dimension *d* over the prime field $\mathbb {F}_{p}$, and let *A* be a nontrivial abelian $p^{\prime }$-subgroup of *H*. The vector space *V* is a completely reducible $\mathbb {F}_{p}A$-module by Maschke’s theorem. We can write $V = V_{1} \oplus {\dots } \oplus V_{k}$, *k* ≤ *d*, where the *V*_*i*_ are irreducible $\mathbb {F}_{p}A$-modules. Write $|V_{i}| = p^{f_{i}}$, $i = 1, \dots , k$, for some integers *f*_*i*_. Let *A*_*i*_ ≤GL(*V*_*i*_) be the group induced on *V*_*i*_ by *A*. Since *A* is abelian, it induces a multiplicative group of a finite field on each *V*_*i*_, so we may assume that $A_{i} \leq \mathbb {F}^{\times }_{p^{f_{i}}}$ for $i = 1, \dots , k$.

We have |*A*_*i*_|≥|*A*|^1/*k*^ for some *i*. Without loss of generality we may assume that for some *j* ≥ 1 we have |*A*_*i*_|≥|*A*|^1/*k*^ for $i = 1, \dots , j$, and |*A*_*i*_| < |*A*|^1/*k*^ for $i = j+1, \dots , k$. Nonzero orbits of *A*_*i*_ on *V*_*i*_ have size equal to |*A*_*i*_|, and in particular, for $i = 1, \dots , j$ nonzero orbits of *A*_*i*_ on *V*_*i*_ have size at least |*A*|^1/*k*^, while for $i = j+1, \dots , k$ orbit sizes are less than |*A*|^1/*k*^.

If the subgroup *A* is normal in *H*, then all $ \mathbb {F}_{p}A $-modules *V*_*i*_, $i = 1, \dots , k$, are isomorphic, in particular, *j* = *k* in this case.

Every vector *v* ∈ *V* can be uniquely written as *v* = *v*_1_ + ⋯ + *v*_*k*_, where *v*_*i*_ ∈ *V*_*i*_, $ i = 1, \dots , k $. We say that *v*_*i*_ is the projection of *v* on *V*_*i*_.

### **Lemma 4.1**

Let *v* be a nonzero vector from *V* and set *Δ* = *v*^*H*^ ∪−*v*^*H*^ ∪{0}. If *s* ≤ *k* − *j*, then there exists *w* ∈ 2^*s*^ ⋅*Δ* such that $w \in V_{1} \oplus {\dots } \oplus V_{k-s} $ and *w* has a nonzero projection on *V*_1_.

### *Proof*

We will prove the statement by induction on *s*. Suppose that *s* = 0. Since *Δ* spans *V*, it cannot lie inside the proper subspace $ V_{2} \oplus {\dots } \oplus V_{k} $. Therefore there exists a vector *w* ∈ 1 ⋅*Δ* such that *w* has a nonzero projection on *V*_1_.

Suppose that *s* > 0, so, in particular, *A* is not normal in *H*. By the inductive hypothesis, there exists some vector *u* ∈ 2^*s*− 1^ ⋅*Δ* lying in $V_{1} \oplus {\dots } \oplus V_{k-s+1} $ and having a nonzero projection on *V*_1_, that is, *u* = *u*_1_ + ⋯ + *u*_*k*−*s*+ 1_ where *u*_*i*_ ∈ *V*_*i*_ for every $ i = 1, \dots , k-s+1 $, and *u*_1_≠ 0. Recall that the length of the *A*-orbit of *u*_*k*−*s*+ 1_ is strictly smaller than |*A*|^1/*k*^, in particular, it is smaller than the length of the *A*-orbit of *u*_1_. Therefore there exists some *a* ∈ *A* with $ {u_{1}^{a}} \neq u_{1} $ and $ u_{k-s+1}^{a} = u_{k-s+1} $. The vector *w* = *u* − *u*^*a*^ has a nonzero projection on *V*_1_ and lies in $ V_{1} \oplus {\dots } \oplus V_{k-s} $. It is left to notice that *w* ∈ 2^*s*− 1^ ⋅*Δ*− 2^*s*− 1^ ⋅*Δ* = 2^*s*^ ⋅*Δ*, where the last equality follows from −*Δ* = *Δ*. The inductive argument is over. □

We need the following result of Cochrane and Cipra.

### **Proposition 4.2**

[8, Theorem 1.2] Let *M* be a subgroup of the multiplicative group $ \mathbb {F}_{q}^{\times } $ of the finite field $ \mathbb {F}_{q} $. If *M* generates $ \mathbb {F}_{q} $ additively and |*M*| > 1, then we have $n \cdot M = \mathbb {F}_{q} $ for every $ n \geq 633 (2(q-1)/|M|)^{\frac {\log 4}{\log |M|}} $.

Recall that *A* induces a group *A*_*i*_ on each *V*_*i*_, $ i = 1, \dots , j $, and |*A*_*i*_|≥|*A*|^1/*k*^. We have $ n_{i} \cdot A_{i} = \mathbb {F}_{p^{f_{i}}} $ for all $ i = 1, \dots , j $ provided that $n_{i} \geq 160 \cdot {(2|V_{i}|)}^{\frac {k \log 4}{\log |A|}}$. Indeed, by Proposition 4.2, this holds for $n_{i} \geq 633\left (\frac {2(|V_{i}|-1)}{|A|^{1/k}}\right )^{\frac {\log 4}{\log (|A|^{1/k})}}$ and we have

$$ 633\left( \frac{2(|V_{i}|-1)}{|A|^{1/k}}\right)^{\frac{\log 4}{\log (|A|^{1/k})}} < 633 \cdot (2|V_{i}|)^{\frac{\log 4}{\log (|A|^{1/k})}} \cdot \frac{1}{4} < 160 \cdot (2|V_{i}|)^{\frac{k \log 4}{\log |A|}}. $$

Let *N*_*i*_ be the lower integer part of $160 \cdot {(2|V_{i}|)}^{\frac {k \log 4}{\log |A|}}$ and set $N = 1 + \max \limits _{i} N_{i} $. By definition, *N* ⋅ *A*_*i*_ = *V*_*i*_ for all $ i = 1, \dots , j $.

Let *v* be an arbitrary vector from $ U = V_{1} \oplus {\dots } \oplus V_{j} $. It has a unique decomposition of the form *v* = *v*_1_ + ⋯ + *v*_*j*_, where *v*_*i*_ ∈ *V*_*i*_, $ i = 1, \dots , j $. Let *l*(*v*) denote the number of nonzero projections of *v*, i.e.

$$ l(v) = |\{ i \in \{ 1, \dots, j \} \mid v_{i} \neq 0 \}|. $$

### **Lemma 4.3**

If *v* ∈ *U*, *v*≠ 0, then *d*(4*N*)^*l*(*v*)+ 1^ ⋅ (*v*^*H*^ ∪{0}) = *V*.

### *Proof*

We use induction on *l*(*v*). Suppose that *l*(*v*) = 1. Then *v* lies in some *V*_*i*_ for $ i \in \{ 1, \dots , j \} $, and hence *N* ⋅ *v*^*A*^ = *V*_*i*_ by the definition of *N*. Therefore *dN* ⋅ (*v*^*H*^ ∪{0}) = *V* by Lemma 2.2.

Suppose that *l*(*v*) > 1 and *v*_*i*_≠ 0 for some $ i \in \{ 1, \dots , j \} $. As $ N \cdot {v_{i}^{A}} = V_{i} $, the projection of *N* ⋅ (*v*^*A*^ ∪{0}) on *V*_*i*_ is equal to *V*_*i*_. In particular, $ |N \cdot (v^{A} \cup \{0\})| \geq p^{f_{i}} $.

If $ |2N \cdot (v^{A} \cup \{0\})| = p^{f_{i}} $, then 2*N* ⋅ (*v*^*A*^ ∪{0}) = *N* ⋅ (*v*^*A*^ ∪{0}) and *N* ⋅ (*v*^*A*^ ∪{0}) is a subgroup of *V*. Therefore *dN* ⋅ (*v*^*H*^ ∪{0}) = *V* by Lemma 2.2.

Assume that $|2N \cdot (v^{A} \cup \{0\})| > p^{f_{i}}$. There exist two distinct vectors $u, u^{\prime } \in 2N \cdot (v^{A} \cup \{0\})$ with equal projections on *V*_*i*_, i.e. $u_{i} = u_{i}^{\prime }$. Since the projection of 2*N* ⋅ (*v*^*A*^ ∪{0}) on *V*_*i*_ is equal to *V*_*i*_, there exists a vector *w* ∈ 2*N* ⋅ (*v*^*A*^ ∪{0}) with projection − *u*_*i*_ on *V*_*i*_. At least one of the vectors *u* + *w* or $u^{\prime }+w$ is nonzero; without loss of generality, *u* + *w*≠ 0. Since *u* + *w* has zero projection on *V*_*i*_, we have *l*(*u* + *w*) < *l*(*v*). As

$$ u + w \in 4N \cdot (v^{A} \cup \{0\}) \subseteq 4N \cdot (v^{H} \cup \{0\}), $$

the inductive hypothesis gives

$$ \begin{array}{@{}rcl@{}} V = d(4N)^{l(u + w) + 1} \cdot ((u+w)^{H} \cup \{0\}) &\subseteq& d(4N)^{l(u+w) + 1} \cdot 4N \cdot (v^{H} \cup \{0\})\\ &\subseteq& d(4N)^{l(v) + 1} \cdot (v^{H} \cup \{0\}) \end{array} $$

and the claim is proved.□

Assume first that *A* is normal in *H*. In this case *j* = *k* and *U* = *V*. Each *V*_*i*_ has equal size and |*V*_*i*_|^*k*^ = |*V* |. Let *v* be an arbitrary nonzero vector in *V*. We have *l*(*v*) ≤ *k* ≤ *d*, therefore

$$ \begin{array}{@{}rcl@{}} \overrightarrow{\text{diam}}(V,H) &\leq& d(4N)^{k+1} \leq d (640 \cdot {(2^{k}|V|)}^{\frac{\log 4}{\log |A|}} + 4)^{k+1}\\ &<& d \cdot 644^{k+1} \cdot {(2^{k}|V|)}^{\frac{(k+1)\log 4}{\log |A|}} < d \cdot 2576^{(k+1)k} \cdot {|V|}^{\frac{(k+1)\log 4}{\log |A|}} \end{array} $$

by Lemma 4.3 and Eq. (1). Thus the second part of Theorem 1.3 is proved.

Assume now that *A* is not necessarily a normal subgroup of *H*. Let *v* be an arbitrary nonzero vector in *V*. By Lemma 4.1, we have a nonzero vector *w* in *U* such that *w* ∈ 2^*k*− 1^ ⋅ (*v*^*H*^ ∪−*v*^*H*^ ∪{0}). Thus

$$ V = d (4N)^{l(w)+1} \cdot (w^{H} \cup \{ 0 \}) \subseteq d (4N)^{l(w)+1} \cdot 2^{k-1} \cdot (v^{H} \cup -v^{H} \cup \{ 0\}) $$

by Lemma 4.3. It follows from Eq. (2) that

$$ \text{diam}(V,H) \leq d \cdot (4N)^{k+1} \cdot 2^{k-1}. $$

Observe that $N \leq 1 + 160 \cdot (2|V|)^{\frac {k \log 4}{\log |A|}} \leq 161 \cdot (2|V|)^{\frac {k \log 4}{\log |A|}}$. Therefore

$$ \begin{array}{@{}rcl@{}} \text{diam}(V,H) &\leq& 2d \cdot 8^{k} \cdot 161^{k+1} \cdot {(2|V|)}^{\frac{k (k+1)\log 4}{\log |A|}}\\ &<& 322 d \cdot 1288^{k} \cdot {(2|V|)}^{\frac{k (k+1)\log 4}{\log |A|}} < 322 d \cdot 144^{k(k+1)} \cdot {|V|}^{\frac{k (k+1)\log 4}{\log |A|}}, \end{array} $$

where the last inequality uses the facts that |*A*|≥ 2 and *k* ≥ 1. The first part of Theorem 1.3 is proved.

## Proof of Theorem 1.1

Recall that an element of a finite group is called a $p^{\prime }$*-element* if its order is not divisible by the prime *p*. The following lemma shows that finite simple groups of Lie type contain elements of large order.

### **Lemma 5.1**

Any finite simple group of Lie type in characteristic *p* contains a $p^{\prime }$-element of order at least *p*^1/5^.

### *Proof*

Assume first that *p* ≥ 7. Let *S* be a finite simple group of Lie type in characteristic *p*. Let *K* be the corresponding universal version, and recall that $S \simeq K/Z(K)$. By [10, Theorem 2.4.7 (d)], the Cartan subgroup of *K* contains a cyclic subgroup of order *p* − 1, so let *C* be the image of that subgroup in *S*. Clearly *C* is a cyclic group of order not divisible by *p*, and |*C*|≥ (*p* − 1)/|*Z*(*K*)|.

Unless *S* has type *A*_*l*_ or ^2^*A*_*l*_, [10, Table 2.2] gives us |*Z*(*K*)|≤ 4, which proves the lemma in this case, since (*p* − 1)/4 ≥ *p*^1/5^. If *S* has type *A*_*l*_ or ^2^*A*_*l*_, then it has a $p^{\prime }$-element of order at least (*p* − 1)/2, see, for instance, [5, Corollary 3].

Assume that *p* ≤ 5. The order of *S* has at least three distinct prime divisors by Burnside’s theorem. It follows that *S* must have a $p^{\prime }$-element of order at least 3 > 5^1/5^ ≥ *p*^1/5^. □

We need the following modular analogue of Jordan’s theorem on linear groups. The original result was proved by Weisfeiler [22, 23] with the use of the classification of finite simple groups, but we use a classification-free version due to Larsen and Pink.

### **Proposition 5.2**

[13, Theorem 0.2] For every *d* there exists a constant *J*(*d*) depending only on *d* such that any subgroup *H* of GL(*d*,*p*), *p* prime, possesses normal subgroups *P* ≤ *B* ≤ *E* such that

1. |*H* : *E*|≤ *J*(*d*).
2. Either *E* = *B*, or *E*/*B* is a direct product of finite simple groups of Lie type in characteristic *p*.
3. *B*/*P* is an abelian $p^{\prime }$-group.
4. *P* is a (possibly trivial) *p*-group.

As a consequence of the above two results, we show that irreducible linear groups contain large abelian $p^{\prime }$-subgroups.

### **Corollary 5.3**

Let *H* be a subgroup of GL(*d*,*p*) acting irreducibly on the vector space of dimension *d* over $\mathbb {F}_{p}$, where *p* is prime.

1. If *H* has a composition factor isomorphic to a finite simple group of Lie type in characteristic *p*, then *H* contains a $p^{\prime }$-element of order at least *p*^1/5^.
2. If *H* does not have a composition factor isomorphic to a finite simple group of Lie type in characteristic *p*, and |*H*|≥ *J*(*d*)^2^, where *J*(*d*) is as in Proposition 5.2, then *H* contains an abelian normal $p^{\prime }$-subgroup of order at least |*H*|^1/2^.

### *Proof*

Assume that *H* has a composition factor *S* isomorphic to a finite simple group of Lie type in characteristic *p*. The group *S* contains a $p^{\prime }$-element *x* of order at least *p*^1/5^. The preimage of *x* in *H* has order divisible by a number coprime to *p* and at least *p*^1/5^. This proves (1).

Assume that *H* does not have a composition factor isomorphic to a finite simple group of Lie type in characteristic *p*. We use Proposition 5.2 and its notation. Since *H* acts irreducibly on the underlying module of dimension *d* over $\mathbb {F}_{p}$, the normal *p*-subgroup *P* of *H* is trivial by Clifford’s theorem. We have *E* = *B* by assumption. Since *B* is an abelian normal $p^{\prime }$-subgroup, we are finished if |*B*|≥|*H*|^1/2^. If |*B*| < |*H*|^1/2^, then |*H*|^1/2^ < |*H* : *B*|≤ *J*(*d*), contradicting our assumption |*H*|≥ *J*(*d*)^2^. This proves (2).□

We return to the proof of Theorem 1.1. Suppose that *H* contains a composition factor isomorphic to a finite simple group of Lie type in characteristic *p*. Then Corollary 5.3 (1) and Theorem 1.3 give us

$$ \text{diam}(V, H) < 322 \cdot d \cdot 144^{d(d+1)} \cdot |V|^{\frac{d(d+1)\log 4}{\log |A|}}, $$

where *A* is an abelian $p^{\prime }$-subgroup of *H* with |*A*|≥ *p*^1/5^. Thus

$$ \text{diam}(V, H) < 322 \cdot d \cdot 144^{d(d+1)} \cdot 4^{5d^{2}(d+1)} \leq 2^{22d^{3}}, $$

where the last inequality holds for all *d* ≥ 2.

If *H* does not contain a composition factor isomorphic to a finite simple group of Lie type in characteristic *p*, and |*H*|≥ *J*(*d*)^2^, then Corollary 5.3 (2) and Theorem 1.3 imply

$$ \overrightarrow{\text{diam}}(V, H) < d \cdot 2576^{d(d+1)} \cdot |V|^{\frac{(d+1) \log 4}{\log |A| }}, $$

where *A* is a normal abelian $p^{\prime }$-subgroup of *H* with |*A*|≥|*H*|^1/2^. Hence

$$ \overrightarrow{\text{diam}}(V, H) < d \cdot 2576^{d(d+1)} \cdot |V|^{\frac{2(d+1) \log 4}{\log |H| }} \leq 2^{18d^{2}} \cdot |V|^{\frac{d \log 64}{\log |H| }}, $$

where the last inequality holds for all *d* ≥ 2. If *d* = 1, then $V = \mathbb {F}_{p}$ and *H* is a multiplicative subgroup of $\mathbb {F}_{p}^{\times }$. By Proposition 4.2, we have

$$ \overrightarrow{\text{diam}}(V, H) \leq 633 \cdot |V|^{\frac{\log 4}{\log |H|}} < 2^{18} \cdot |V|^{\frac{\log 64}{\log |H|}}, $$

so the claimed inequality holds in this case as well.

## Proof of Corollary 1.2

The lower bound is the first inequality from Proposition 3.1.

Let *J*(*d*) be the function from Theorem 1.1. Suppose that |*H*| < *J*(*d*)^2^. Then $\frac {\log |V|}{\log |H|} \geq \frac {d \log p}{\log J(d)^{2}}$ and taking *f*(*d*) ≥ *J*(*d*)^4^ we have

$$ f(d)^{\frac{\log |V|}{\log |H|}} \geq (J(d)^{2})^{\frac{2d \log p}{\log J(d)^{2}}} = p^{2d} \geq p \cdot d. $$

By Proposition 3.2, the orbital diameter is always at most *p* ⋅ *d*, so we are done.

Now assume that |*H*|≥ *J*(*d*)^2^. Recall that $|H| \leq |\text {GL}(V)| \leq p^{d^{2}}$, hence $\log |V| / \log |H| \geq 1/d$. Now taking

$$ f(d) \geq \left( \max \{ 2^{22d^{3}}, 2^{18d^{2}} \cdot 64^{d} \}\right)^{d} $$

and applying Theorem 1.1 proves the claim.

## Affine Groups with Large Orbital Diameters

In this section we provide several series of groups with orbital graphs of large diameter.

Let *K* be a nontrivial subgroup of $\mathbb {F}_{p}^{\times }$, where *p* is an odd prime. Let *S* be a transitive permutation group of degree *d* and let *H* = *K* ≀ *S* be a wreath product acting (linearly) imprimitively on $V = \mathbb {F}_{p}^{d}$. By [21, Chapter IV, Section 15, Lemma 4], since *p* is odd and *K* is nontrivial, the group *H* acts irreducibly on *V*.

If {*v*_1_,…,*v*_*d*_} is the standard basis of *V*, then the orbit of *v*_1_ under *H* is ${v_{1}^{H}} = {v}^{K}_{1} \cup {\cdots } \cup {v}^{K}_{d}$. Therefore

$$ m \cdot \left( {v_{1}^{H}} \cup \{0\}\right) = \bigcup_{\substack{m_{1}, \dots, m_{d} \geq 0,\\m_{1} + {\cdots} + m_{d} \leq m}} \left( m_{1} \cdot {v_{1}^{K}} + {\dots} + m_{d} \cdot {v_{d}^{K}}\right), $$

for any *m* ≥ 1 and hence $\overrightarrow {\text {diam}}(V,H) \geq \overrightarrow {\text {diam}}(\mathbb {F}_{p}, K) \cdot d$ by Eq. (1). As $\mathbb {F}_{p}^{\times } \cap H = K$, Proposition 3.2 implies that $\overrightarrow {\text {diam}}(V,H) \leq \overrightarrow {\text {diam}}(\mathbb {F}_{p}, K) \cdot d $. Therefore we proved

### **Proposition 7.1**

Let *p* be an odd prime, and let *K* be a nontrivial subgroup of $\mathbb {F}_{p}^{\times }$. If *S* is a transitive permutation group of degree *d*, then the (linearly) imprimitive wreath product *H* = *K* ≀ *S* acts irreducibly on *V* and $\overrightarrow {\text {diam}}(V, H) = \overrightarrow {\text {diam}}(\mathbb {F}_{p}, K) \cdot d$.

Clearly, $\text {diam}(V, H) = \text {diam}(\mathbb {F}_{p}, K \langle -1 \rangle ) \cdot d$. By taking appropriate *K* it easily follows that the inequalities on undirected diameter presented in Proposition3.2 are sharp.

Let *r* ≥ 5 be an integer. In the second example *H* is the alternating group Alt(*r*) and *p* is an odd prime not dividing *r*. Let {*v*_1_,…,*v*_*r*_} be the basis of the natural permutation module of *H* over the field $\mathbb {F}_{p}$. This module has a proper submodule *V* which consists of vectors $v = {\sum }_{i=1}^{r} \lambda _{i} v_{i}$, where each *λ*_*i*_ is an element of $\mathbb {F}_{p}$, such that ${\sum }_{i=1}^{r} \lambda _{i} = 0$. The module *V* is irreducible by [12, Lemma 5.3.4] and has dimension *d* = *r* − 1 over $\mathbb {F}_{p}$. We will prove that diam(*V*,*H*) ≥ (*p* − 1)*d*/4.

Consider the *H*-orbit ${\varDelta } = \{\pm (v_{i} - v_{j}) ~|~ 1 \leq i < j \leq r \} \subseteq V$. Let *m* be the smallest positive integer such that *m* ⋅ (*Δ* ∪{0}) = *V*. Since this is a lower bound for diam(*V*,*H*) by Eq. (2), it is sufficient to show that *m* ≥ ((*p* − 1)/2) ⋅ *d*/2.

Observe that $m = \max \limits _{v \in V} \{ m(v) \}$ where *m*(*v*) is the smallest positive integer such that *v* ∈ *m*(*v*) ⋅ (*Δ* ∪{0}). Denote the elements of $\mathbb {F}_{p}$ by 0,± 1,…,±(*p* − 1)/2. Let $v = {\sum }_{k=1}^{r} \lambda _{k} v_{k}$ where each *λ*_*k*_ is in $\mathbb {F}_{p}$. We claim that $2 m(v) \geq {\sum }_{k=1}^{r} |\lambda _{k}|$. This would finish the proof of the lower bound, as one can take *λ*_*k*_ = (*p* − 1)/2 for $ k = 1, \dots , r-1 $.

We prove the claim by induction on *t* = *m*(*v*). This is clear for *t* ≤ 1. Assume that *t* ≥ 2 and that the claim is true for *t* − 1. Let $v = {\sum }_{k=1}^{m(v)} \delta _{k}$ where each *δ*_*k*_ ∈*Δ*. Let *δ*_*m*(*v*)_ = *v*_*i*_ − *v*_*j*_ for some distinct *i* and *j* from {1,…,*r*}. Let $v^{\prime } = {\sum }_{k=1}^{m(v)-1} \delta _{k}$. This is $v^{\prime } = v - \delta _{m(v)} = ({\sum }_{i=1}^{r} \lambda _{k} v_{k} ) + v_{j} - v_{i}$. By the induction hypothesis, $2 m(v^{\prime }) \geq ({\sum }_{k=1}^{r} |\lambda _{k}|) - |\lambda _{i}| - |\lambda _{j}| + |\lambda _{i}-1| + |\lambda _{j}+1|$. On the other hand, $2 m(v) - 2 \geq 2 m(v^{\prime })$. Since 1 −|*λ*_*i*_| + |*λ*_*i*_ − 1|≥ 0 and 1 −|*λ*_*j*_| + |*λ*_*j*_ + 1|≥ 0, the result follows.

### **Proposition 7.2**

For every *d* ≥ 4 and every odd prime *p* not dividing *d* + 1 there exists a nonabelian simple group *H* with diam(*V*,*H*) ≥ (*p* − 1)*d*/4.

In Corollary 1.2 it was shown that there exists a function *f*(*d*) depending on *d* only such that $\text {diam}(V, H) \leq f(d)^{\frac {\log |V|}{\log |H|}}$ for any irreducible subgroup *H* ≤GL(*V* ), where *V* has dimension *d* over $\mathbb {F}_{p}$. Proposition 7.1 (and also Proposition 7.2) implies that *f*(*d*) must depend on *d* at least quasipolynomially. For example, for *p* = 3 and *d* ≥ 4 let *S* be the symmetric group Sym(*d*) and *K* = 〈− 1〉. Then |*H*| = |*K* ≀ *S*| = 2^*d*^ ⋅ *d*! and $\log |H| \geq d \log (d/e)$. We have diam(*V*,*H*) = *d*(*p* − 1)/2 = *d* hence

$$ d \leq f(d)^{\frac{\log |V|}{\log |H|}} \leq f(d)^{\frac{\log 3}{\log (d/e)}}. $$

Thus $ d^{\frac {\log (d/e)}{\log 3}} \leq f(d) $, as claimed.

In our third example, let *q* and *d* be integers with *q* ≥ 2 and *d* ≥ 3. Zsigmondy’s theorem [24] states that, provided (*q*,*d*)≠(2,6), there is a prime *r* dividing *q*^*d*^ − 1 but not dividing *q*^*i*^ − 1 for all *i* < *d*. Such a prime *r* is called a *Zsigmondy prime* and is denoted by *q*_*d*_. There may be several Zsigmondy primes *q*_*d*_ for given *d* and *q*. It is easy to see that *q*_*d*_ must be congruent to 1 modulo *d*. If *d* + 1 is prime then *d* + 1 is a Zsigmondy prime *q*_*d*_ whenever the multiplicative order of *q* modulo *d* + 1 is *d*. For a given prime *d* + 1 there are infinitely many primes *q* with this property by Dirichlet’s prime number theorem.

Let *p* and *d* be such that *d* + 1 is an odd Zsigmondy prime *p*_*d*_. Set $V = \mathbb {F}_{p}^{d}$ and let *h* be an element of GL(*V* ) of order *d* + 1. Since 〈*h*〉 is a cyclic group of order coprime to *p*, the $\mathbb {F}_{p}\langle h \rangle $-module *V* is completely reducible by Maschke’s theorem. Moreover, since the order of 〈*h*〉 is a Zsigmondy prime, *V* must be irreducible.

Let *Δ* be an orbit of 〈*h*〉. It must have size *d* + 1 and it must contain a basis of *V*; set *Δ* = {*v*_1_,…,*v*_*d*_,*v*}, where $v_{1}, \dots , v_{d}$ are linearly independent. Since *Δ* is an orbit, *h* preserves the sum over all vectors in *Δ*:

$$ (v_{1} + {\cdots} + v_{d} + v)^{h} = v_{1} + {\cdots} + v_{d} + v, $$

and as 〈*h*〉 acts irreducibly on *V*, this sum must be zero. Therefore $v = - {\sum }_{j=1}^{d} v_{j}$.

Let *H* be the cyclic group 〈*h*〉〈− 1〉. By the previous paragraph every orbit *Δ* of *H* has the form $\{\pm v_{1} \} \cup {\cdots } \cup \{ \pm v_{d} \} \cup \{ \pm ({\sum }_{i=1}^{d} v_{i}) \}$ where {*v*_1_,…,*v*_*d*_} is a basis for *V*. Let *ℓ* be the smallest positive integer such that *ℓ* ⋅ (*Δ* ∪{0}) = *V*. Since *Δ* = −*Δ*, this is equal to diam(*V*,*H*) by Eq. 2. We proceed to show that (*p* − 1)*d*/4 ≤ *ℓ* ≤ (*p* − 1)(*d* + 1)/4, provided that *p* is odd.

Assume that *p* is odd. Let *x*, 0 = *x*_0_, *x*_1_,…,*x*_*d*_ denote elements of $\mathbb {F}_{p}$ where we will allow *x*, *x*_1_,…,*x*_*d*_ to vary. For two elements *f*_1_ and *f*_2_ of $\mathbb {F}_{p}$ let *D*(*f*_1_,*f*_2_) be their distance defined to be the smallest integer *m* for which *f*_1_ ∈ *f*_2_ + *m*{± 1,0}. The smallest integer *m* for which the vector ${\sum }_{i=1}^{d} x_{i} v_{i}$ is contained in *m* ⋅ (*Δ* ∪{0}) is $\min \limits _{x \in \mathbb {F}_{p}} \{ {\sum }_{i=0}^{d} D(x_{i} , x) \}$. We have

$$ \begin{array}{@{}rcl@{}} \min_{x \in \mathbb{F}_{p}}\left\{\sum\limits_{i=0}^{d} D(x_{i},x)\right\} &\geq& \min_{f_{d/2} \in \mathbb{F}_{p}} \{ D(x_{d/2}, f_{d/2}) \} + \sum\limits_{i=0}^{\frac{d}{2}-1} \min_{f_{i} \in \mathbb{F}_{p}} \{ D(x_{i},f_{i}) + D(x_{d-i},f_{i}) \} \\ &=& \sum\limits_{i=0}^{\frac{d}{2}-1} \min_{f_{i} \in \mathbb{F}_{p}} \{ D(x_{i},f_{i}) + D(x_{d-i},f_{i}) \} = \sum\limits_{i=0}^{\frac{d}{2}-1} D(x_{d-i}, x_{i}). \end{array} $$

It follows that

$$ \ell = \max_{\substack{x_{1}, {\ldots} , x_{d} \in \mathbb{F}_{p}\\ x_{0} = 0}} \left\{\min_{x \in \mathbb{F}_{p}} \left\{\sum\limits_{i=0}^{d} D(x_{i}, x)\right\}\right\} \geq \max_{\substack{x_{1}, {\ldots} , x_{d} \in \mathbb{F}_{p}\\ x_{0} = 0}} \sum\limits_{i=0}^{\frac{d}{2}-1} D(x_{d-i}, x_{i}) \geq \frac{(p-1)d}{4}. $$

On the other hand,

$$ \min_{x \in \mathbb{F}_{p}} \left\{\sum\limits_{i=0}^{d} D(x_{i} , x)\right\} \leq \min \left\{\sum\limits_{i=0}^{d} D(x_{i} , 0), \sum\limits_{i=0}^{d} D(x_{i} , (p-1)/2) \right\}. $$

Without loss of generality we can renumber $ x_{0}, \dots , x_{d} $ in such a way that for some *z* and *k* we have *x*_0_ = ⋯ = *x*_*z*_ = 0, 1 ≤ *x*_*z*+ 1_ ≤⋯ ≤ *x*_*k*_ ≤ (*p* − 1)/2 and − 1 ≥ *x*_*k*+ 1_ ≥⋯ ≥ *x*_*d*_ ≥−(*p* − 1)/2. With this assumption,

$$ \begin{array}{@{}rcl@{}} {\sum}_{i=0}^{d} D(x_{i} , 0) &=& \left( \sum\limits_{i=z+1}^{k} x_{i}\right) - \left( \sum\limits_{i=k+1}^{d} x_{i}\right),\\ \sum\limits_{i=0}^{d} D(x_{i} , (p-1)/2) &=& \frac{(p-1)(d+1)}{2} - \left( \sum\limits_{i=z+1}^{k} x_{i}\right) + \left( \sum\limits_{i=k+1}^{d} x_{i}\right). \end{array} $$

It follows that

$$ \ell = \max_{\substack{x_{1}, {\ldots} , x_{d} \in \mathbb{F}_{p}\\ x_{0} = 0}} \left\{\min_{x \in \mathbb{F}_{p}} \left\{\sum\limits_{i=0}^{d} D(x_{i}, x)\right\}\right \} \leq \frac{(p-1)(d+1)}{4}. $$

We summarize the discussed example in the following

### **Proposition 7.3**

There are infinitely many positive integers *d* and, for each such *d*, infinitely many primes *p* and cyclic groups *H* for which (*p* − 1)*d*/4 ≤diam(*V*,*H*) ≤ (*p* − 1)(*d* + 1)/4.

Note that the argument from Proposition 7.3 can be used to obtain another proof of the lower bound in Proposition 7.2. In the notation of the second example, take an orbit *Δ* = {*v*_*i*_ − *u*∣1 ≤ *i* ≤ *r*} where $u = \frac {1}{r} {\sum }_{i = 1}^{r} v_{i}$; recall that *H* = Alt(*r*) permutes $v_{1}, \dots , v_{r}$. One can check that *v*_*r*_ − *u* = −(*v*_1_ − *u*) −⋯ − (*v*_*r*− 1_ − *u*), hence *Δ* = {*e*_1_,…,*e*_*r*− 1_,−(*e*_1_ + ⋯ + *e*_*r*− 1_)} where *e*_*i*_ = *v*_*i*_ − *u* for all *i* with 1 ≤ *i* ≤ *r* − 1 and {*e*_1_,…,*e*_*r*− 1_} is a basis for *V*. If we disregard the group *H*, we arrive at the same orbital graph as in Proposition 7.3, giving the expected (*p* − 1)*d*/4 ≤diam(*V*,*H*) lower bound.
